# JDP2 overexpression provokes cardiac dysfunction in mice

**DOI:** 10.1038/s41598-018-26052-w

**Published:** 2018-05-16

**Authors:** Jacqueline Heger, Julia Bornbaum, Alona Würfel, Christian Hill, Nils Brockmann, Renáta Gáspár, János Pálóczi, Zoltán V. Varga, Márta Sárközy, Péter Bencsik, Tamás Csont, Szilvia Török, Baktybek Kojonazarov, Ralph Theo Schermuly, Kerstin Böngler, Mariana Parahuleva, Peter Ferdinandy, Rainer Schulz, Gerhild Euler

**Affiliations:** 1 Institute of Physiology, Justus Liebig University, Giessen, Germany; 2 Department of Biochemistry, University of Szeged, Szeged, Hungary; 3 Pharmahungary Group, Szeged, Hungary; 4 Department of Pharmacology and Pharmacotherapy, Semmelweis University, Budapest, Hungary; 5 Universities of Giessen and Marburg Lung Center (UGMLC), Excellence Cluster Cardio-Pulmonary System (ECCPS), Member of the German Center for Lung Research (DZL), Giessen, Germany; 6 Internal Medicine/Cardiology and Angiology, University Hospital of Giessen and Marburg, Location Marburg, Marburg, Germany

## Abstract

The transcriptional regulator JDP2 (Jun dimerization protein 2) has been identified as a prognostic marker for patients to develop heart failure after myocardial infarction. We now performed *in vivo* studies on JDP2-overexpressing mice, to clarify the impact of JDP2 on heart failure progression. Therefore, during birth up to the age of 4 weeks cardiac-specific JDP2 overexpression was prevented by doxycycline feeding in transgenic mice. Then, JDP2 overexpression was started. Already after 1 week, cardiac function, determined by echocardiography, decreased which was also resembled on the cardiomyocyte level. After 5 weeks blood pressure declined, ejection fraction and cardiac output was reduced and left ventricular dilatation developed. Heart weight/body weight, and mRNA expression of ANP, inflammatory marker genes, collagen and fibronectin increased. Collagen 1 protein expression increased, and fibrosis developed. As an additional sign of elevated extracellular matrix remodeling, matrix metalloproteinase 2 activity increased in JDP2 mice. Thus, JDP2 overexpression is deleterious to heart function *in vivo*. It can be concluded that JDP2 overexpression provokes cardiac dysfunction in adult mice that is accompanied by hypertrophy and fibrosis. Thus, induction of JDP2 is a maladaptive response contributing to heart failure development.

## Introduction

One of the major burdens in human health care is the strong prevalence of heart failure which is associated with high mortality rates. Although in the last decade some new therapeutic approaches resulted in a slight increase in survival rates, the 5-year incidence of death from heart failure is still greater than 60%^1^. Therefore, identification of novel therapeutic strategies against heart failure is essential.

One approach is the identification of early protein changes that predict the development of HF after an initial cardiac event. Just recently, Maciejak *et al*.^2^ conducted a study in patients with myocardial infarction in order to identify such early markers for the risk stratification. Blood samples were taken from patients at the time of admission to hospital, as well as one and six months after the initial event, and gene expression was monitored in peripheral blood mononuclear cells (PBMCs). Three genes that were most significantly upregulated only in PBMCs of patients who developed HF, were identified. These genes were formin 1 (FMN1), ribonuclease 1 (RNASE1), and Jun dimerization protein 2 (JDP2). The increased expression of these genes on admission of patients to hospital positively correlated with a decrease in left ventricular function six months after myocardial infarction. Thus, FMN1, RNASE1 and JDP2 expression are promising candidates for the risk stratification of patients to develop HF after myocardial infarction. In addition, the strong correlation between the expressions of these genes with HF development implies their direct involvement in the process of HF.

Here we now focused our study on the role of JDP2 for HF development. JDP2 is a member of the basic leucine zipper (bZIP) protein superfamily of transcription factors. JDP2 was originally isolated based on its association with c-Jun^3^, which is a component of the transcription factor AP-1 (activator protein 1), and ATF2 (activating transcription factor 2)^4^, reviewed by Huang *et al*.^5^. JDP2 is constitutively expressed in all cells tested^6^, and can bind TRE (trans-activator response element) and CRE (cAMP responsive promoter element) DNA elements as homo- or hetero-dimers^3,4^, both of which are binding sites for AP-1. Upon dimerization of JDP2 with c-Jun, DNA binding is potentiated, but AP-1-mediated transcription is inhibited^3^. JDP2 inhibits transcription by multiple mechanisms which include competition for DNA binding sites of positive transcription factors^3^, competition with c-Jun N-terminal kinase phosphorylation^6,7^, promotion of nucleosome assembly activity^8^, and through direct recruitment of multiple members of histone deacetylases (HDACs) to cognate TRE-containing promoters^9^.

Cardiomyocytes isolated from JDP2-overexpressing transgenic mice revealed protection against the induction of hypertrophic growth and apoptosis^10^. These results imply a protective role of JDP2 in heart failure, and the induction of JDP2 after myocardial infarction may be an adaptive process. Another argument for the protective role of JDP2 is the recent finding, that knock out of JDP2 promotes cardiac hypertrophy and dysfunction in response to pressure overload in mice^11^. However, besides these positive effects, JDP2 also has detrimental effects in cardiomyocytes, since the contractile function of ventricular cardiomyocytes isolated from JDP2-overexpressing mice was impaired: While wildtype cardiomyocytes showed enhanced cell shortening as well as faster contraction and relaxation velocities with increasing amounts of the beta adrenoceptor agonist isoprenaline, JDP2-overexpressing cells failed to respond to β-adrenergic stimulation^10^. Due to these contradictory activities of JDP2 in cardiomyocytes, preventing processes of adverse cardiac remodeling, like hypertrophy or apoptosis, while simultaneously reducing their contractile capacity, it remains unclear, if JDP2 overexpression is protective or detrimental for the heart.

So far, the cardiac phenotype of JDP2 mice has been analysed in 4 week old mice. These studies revealed development of bi-atrial dilatation and defects in the conduction system^12^. No overt ventricular heart disease was detected. In histological sections of JDP2-overexpressing hearts, hypertrophic cardiomyocytes were detected in atria, but not in ventricles. However, as heart failure predominantly occurs in the elderly patient, we now determined the role of enhanced JDP2 expression in adult mice hearts. Since the expression of JDP2 in transgenic mice is under the control of a tetracycline-regulated α-MHC promoter, cardiac expression of JDP2 could be repressed by doxycycline feeding during juvenile development. In 4-week-old animals, JDP2 overexpression was started, and the effect of JDP2 on the cardiac phenotype and function was determined one and five weeks after JDP2 overexpression in adult mice.

## Results

### Cardiac function under JDP2 overexpression

**Figure 1:**
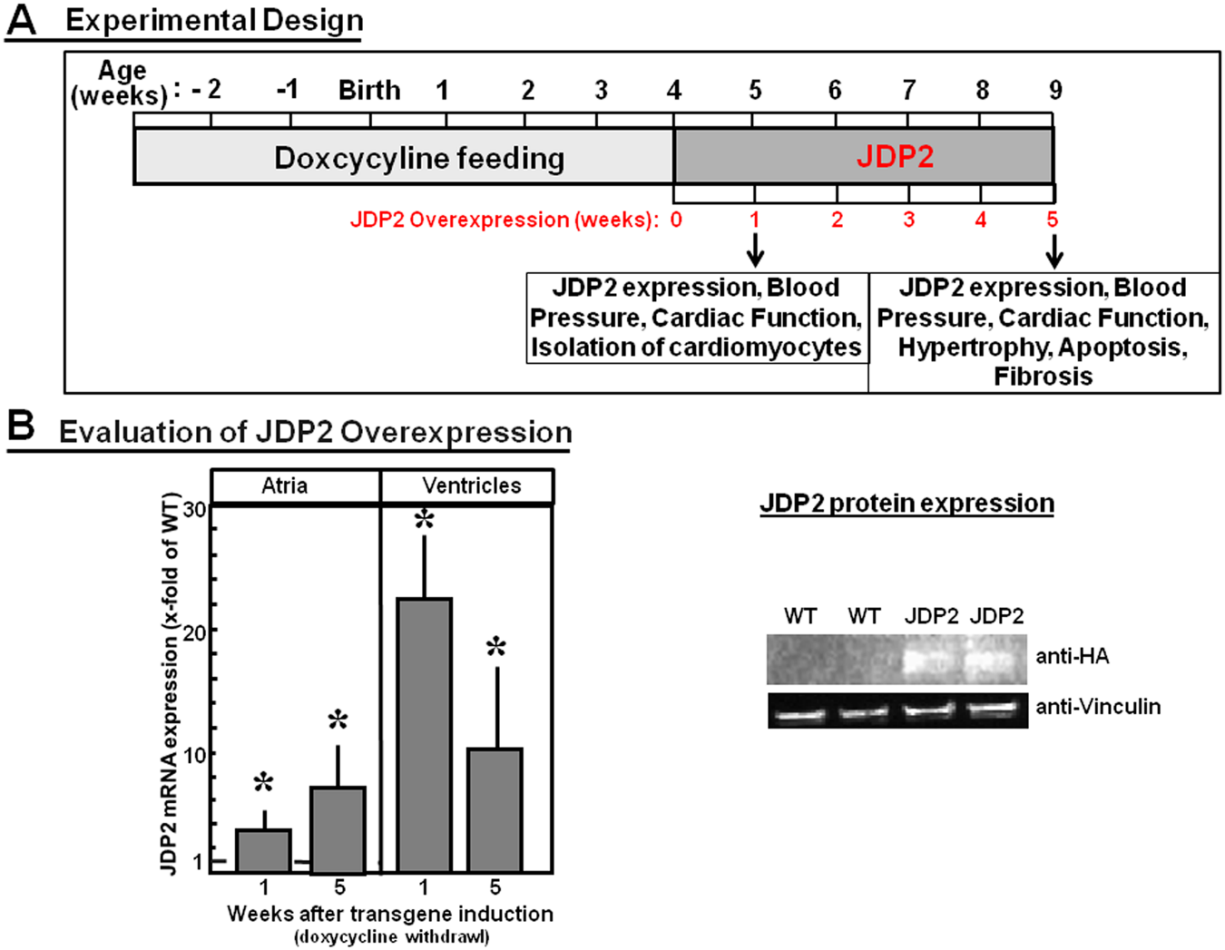
(**A**) Experimental study design. JDP2-overexpressing mice were fed for four weeks with doxycycline (Dox). Then Dox-feeding was stopped and JDP2 was overexpressed for one or five weeks. Parameters related to cardiac performance were analysed. (**B**) Evaluation of JDP2 overexpression. mRNA expressions of JDP2 were evaluated. Data are means ± SD. *Differences from WT-animals with p ≤ 0.05, n = 8. Furthermore, JDP2 protein from 5 weeks overexpressing hearts was detected in western blots by HA antibodies. Vinculin was used as loading control.

Before analysing the phenotype of transgenic JDP2 mice, overexpression of JDP2 in absence of doxycycline feeding was evaluated in atria and ventricles by real time RT-PCR. In the absence of doxycycline JDP2 mRNA expression increased in atria 3.6-fold within one week, and 7.2-fold after five weeks (n = 6, p ≤ 0.05 vs. WT mice). In ventricles JDP mRNA was elevated 22.8-times within one week, and 10-fold within five weeks in absence of doxycycline feeding (n = 8, p ≤ 0.05 vs. WT mice). Furthermore, JDP2 protein expression was detectable in hearts of transgenic animals only (Fig. 1B).

**Figure 2:**
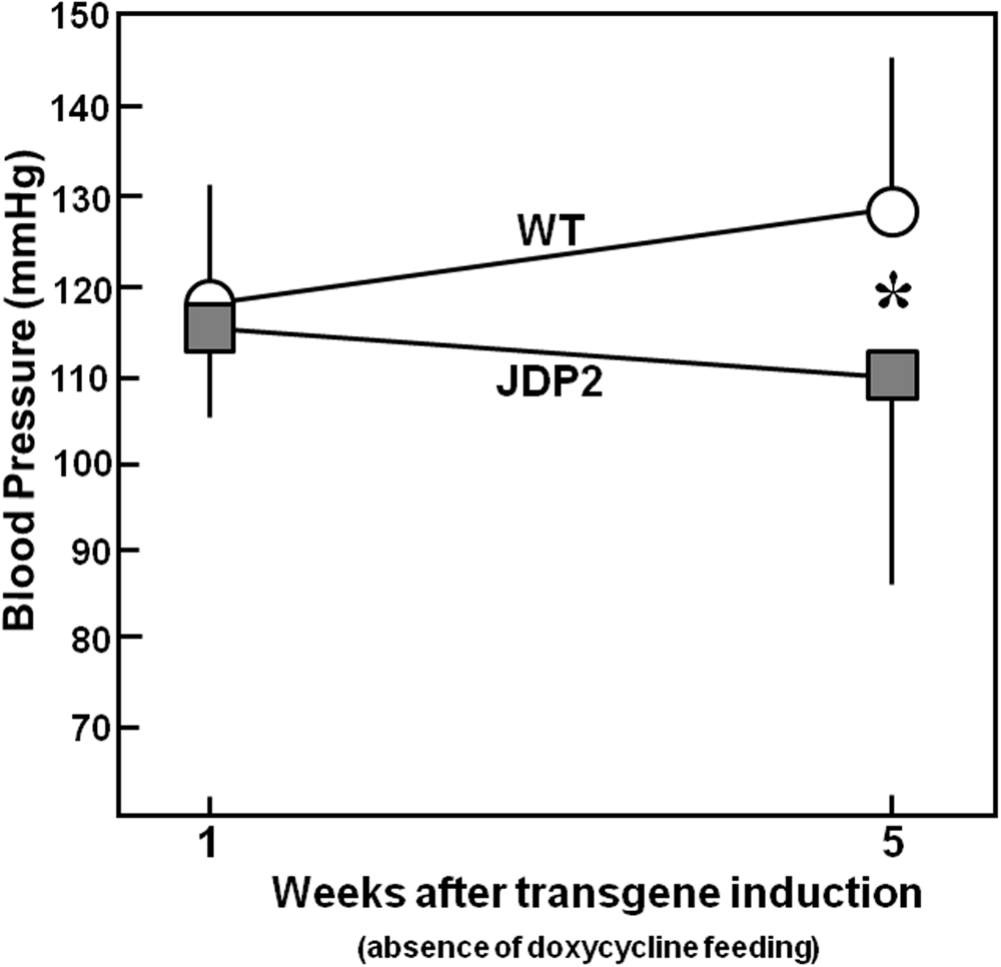
Blood pressure is increased in JDP2 mice. Data are means ± SD. *Differences from WT animals with p ≤ 0.05, n = 15.

Next, we determined the influence of JDP2 overexpression on blood pressure. After one week of JDP2 overexpression blood pressure in WT and JDP2 mice were almost identical. After 5 weeks a significant drop in blood pressure was observed in JDP2 mice when compared to age-matched WT mice (129 ± 16 in WT vs. 110 ± 24 mmHg in JDP2 mice (n = 15 in each group, p ≤ 0.05) (Fig. 2). This decline in blood pressure may indicate a reduced ventricular pump function in JDP2 mice.

For further analysis of cardiac function echocardiography was applied. Overexpression of JDP2 for one week provoked ventricular dysfunction, since cardiac output, fractional shortening, and ejection fraction declined (Table

**Table 1:** Echocardiography data of JDP2-overexpressing and WT mice.

Time of Dox-absence	1 week		5 week	
n	WT	JDP2	WT	JDP2
	15	11	8	11
Left ventricular end diastolic diameter (LVEDD), longitudinal (mm)	3.83 ± 0.60	3.88 ± 0.50	3.38 ± 0.44	3.93 ± 0.51*
Left ventricular end systolic diameter (LVESD), cross sectional (mm)	2.39 ± 0.61	2.85 ± 0.46	1.83 ± 0.51	2.63 ± 0.55*
FS, cross sectional (%)	38.1 ± 7.6	27.4 ± 4.8*	46 ± 9	29 ± 7*
EF, cross sectional (%)	64.6 ± 10.4	58.8 ± 9.3*	66.0 ± 5.4	34.5 ± 7.0*
SV (µl)	49.1 ± 13.0	37.3 ± 6.4	45.7 ± 8.2	28.4 ± 4.1*
CO (ml/min)	23.0 ± 4.8	19.4 ± 3.3*	22.1 ± 5.1	13.6 ± 2.6*

^*^p ≤ 0.05 comparing age-matched WT and JDP2 mice (t-Test).

**Figure 3:**
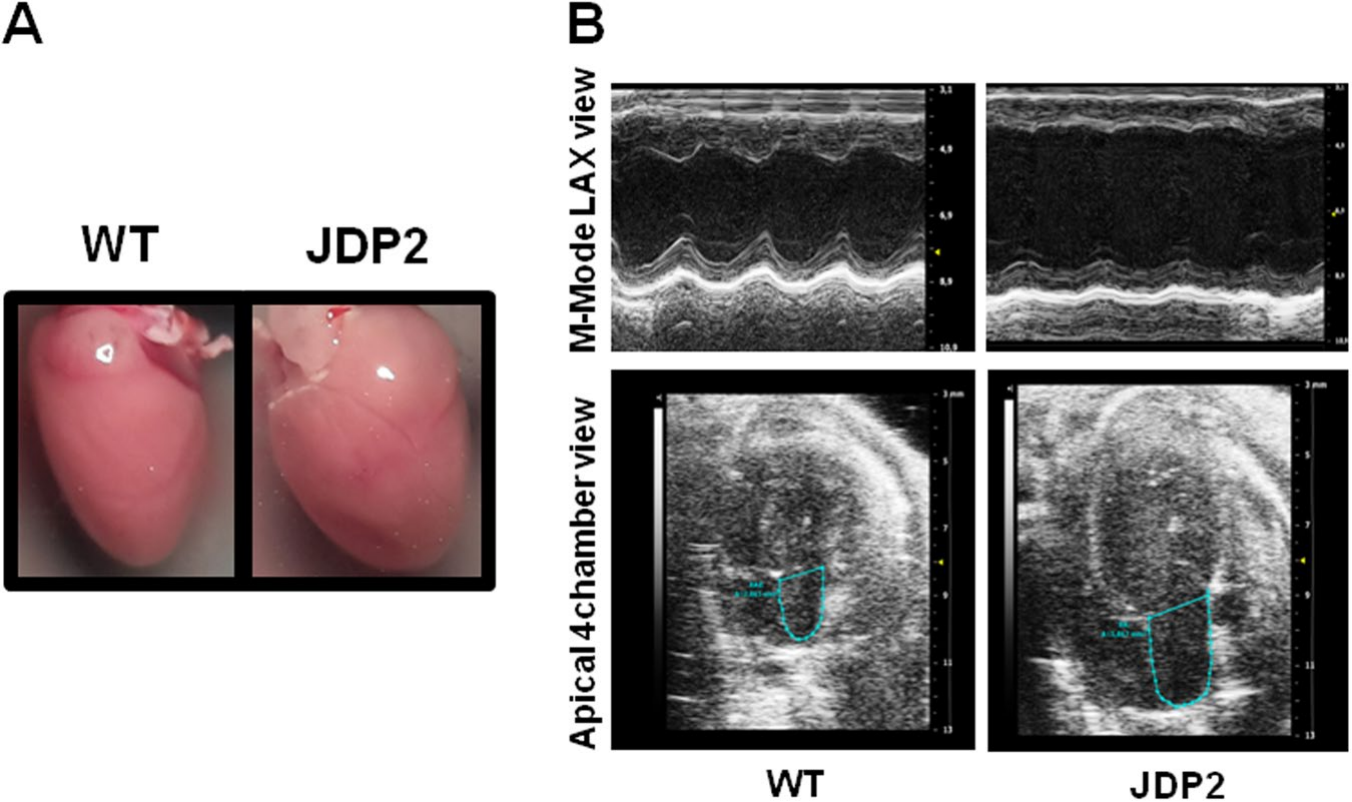
Atrial dilatation after 5 weeks of JDP2 overexpression. (**A)** Micrographs of hearts. (**B**) Echocardiography recordings. Atrial dimensions are indicated.

2). Prolongation of JDP2 overexpression to five weeks aggravated ventricular dysfunction. This was evident by the following parameters: (i) reduction of fractional shortening (FS, cross sectional) from 46 ± 9% to 29 ± 7%, (ii) decline in ejection fraction (EF, cross sectional) from 66 ± 5% to 34 ± 7% (n = 8–11, p ≤ 0.05), and (iii) cardiac output was reduced from 22.1 ± 5.1 to 13.6 ± 2.6 ml/min (Table 2). Furthermore, signs of cardiac dilatation became evident by (i) an increase in left ventricular end diastolic diameter (LVEDD) from 3.38 ± 0.44 mm in WT to 3.93 ± 0.51 mm in JDP2 mice, and (ii) in end systolic diameter (LVESD) (longitudinal) from 1.83 ± 0.51 mm to 2.63 ± 0.55 mm (Table 1). In addition, overexpression of JDP2 for five weeks caused left atrial enlargement to 5.48 ± 0.74 mm^2^ compared to 3.55 ± 0.51 mm^2^ in WT mice (p ≤ 0.05, n = 6–10) (Fig. 3A,B). Atrial dilation was not observed after 1 week of JDP2 overexpression. All together, these analyses demonstrate development of heart failure under prolonged JDP2 overexpression.

### Short-term JDP2 overexpression impairs contractile function of isolated cardiomyocytes

As we have previously demonstrated, life-long JDP2 overexpression reduces contractile performance in isolated cardiomyocytes^13^. This could explain the impaired cardiac function that we observe here in mice overexpressing JDP2 for five weeks. However, we could not exclude that during lifelong overexpression other secondary effects provoked contractile impairments in isolated cardiomyocytes. Therefore, we conducted additional measurements on mice that overexpressed JDP2 for only one week, as these mice already presented reduced cardiac function in echocardiography (Table 2).

**Figure 4:**
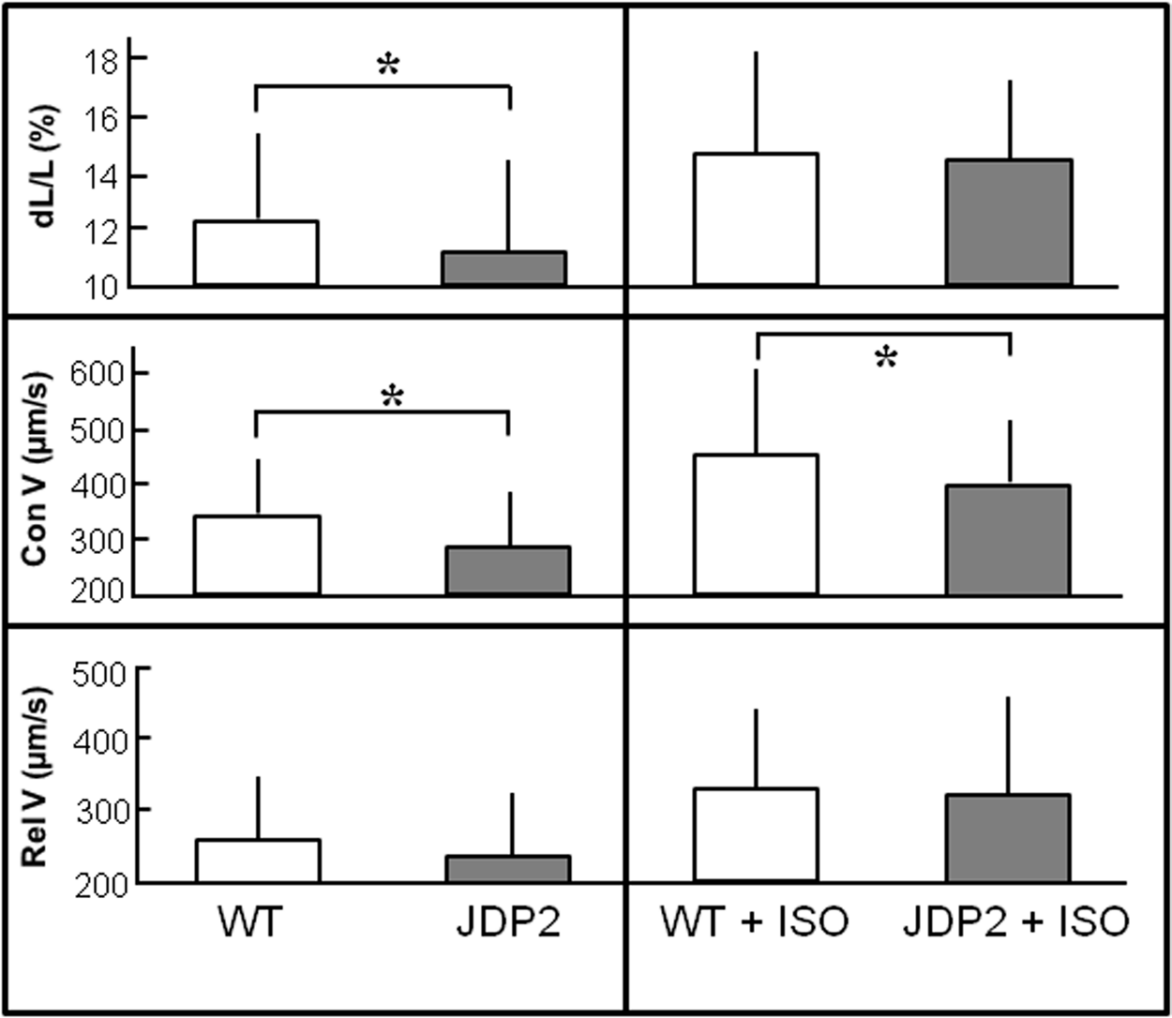
JDP2 overexpression impairs contractile function in ventricular cardiomyoycytes. Cardiomyocytes were isolated from mice one week after start of JDP2 overexpression and from age matched WT animals. Contractions were analysed at 2 Hz in absence or presence of 10 nM isoprenaline (ISO). Data are means ± SD of 100 cells from 4 independent culture preparations. *Differences between WT and JDP2 overexpressing cardiomyocytes with p ≤ 0.05.

Cardiomyocytes were isolated from these mice, and contractile performance was determined under electrical stimulation at 2 Hz. Relative cell shortening (dL/L) was reduced in JDP2-overexpressing cardiomyocytes (11.3 ± 3.3% vs. 12.2 ± 3.4% in WT cells, n = 4, p ≤ 0.05), as well as contraction velocity was impaired (292 ± 107 µm/s in JDP2 vs. 332 ± 130 µm/s in WT cells, n = 4, p ≤ 0.05) (Fig. 4). Under β-adrenergic stimulation with isoprenaline (10 nM) relative cell shortening increased to the same extent in cardiomyocytes of WT and JDP2 mice, but the increase in contraction velocity under isoprenaline was smaller in JDP2 mice (407 ± 117 µm/s vs. 456 ± 148 µm/s in WT, n = 4, p ≤ 0.05) (Fig. 4). Interestingly, expression levels of the calcium pump SERCA was reduced by 46% in hearts of JDP2 mice (n = 6, p ≤ 0.05 vs. WT).

### Cardiac apoptosis, hypertrophy, fibrosis, and inflammation under JDP2 overexpression

**Figure 5:**
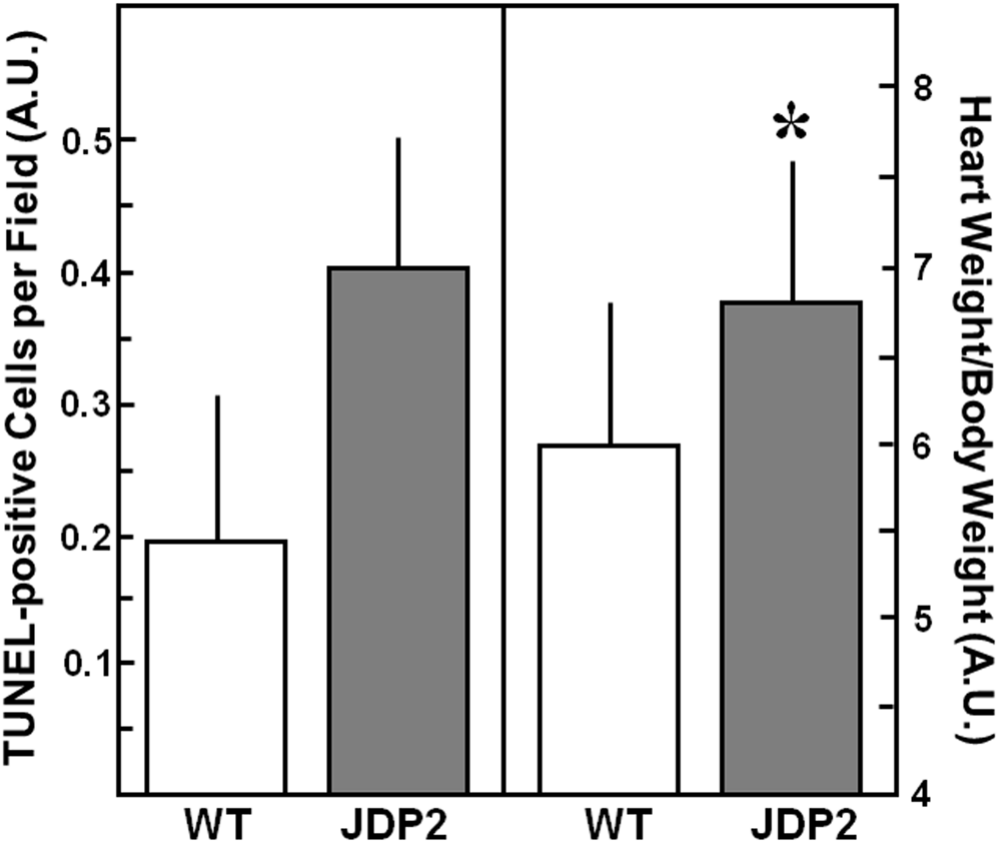
Apoptosis and Hypertrophy increases in JDP2 mice. Hearts were extracted from mice five weeks after start of JDP2 overexpression and from age matched WT animals. Apoptosis was detected by TUNEL-assay (n = 5). As hypertrophic parameter HW to BW ratio was determined (n = 15–22). Data are means ± SD. *Differences from WT animals with p ≤ 0.05.

In order to analyse, if the functional impairment goes along with processes of apoptosis or hypertrophy, we performed TUNEL assay and determined heart to body weight ratio in mice overexpressing JDP2 for five weeks. TUNEL assay revealed a slight, but non-significant increase in apoptotic cells in JDP2 mice: 0.23 ± 0.10 TUNEL-positive cells per field were found in WT mice vs. 0.38 ± 0.11 in mice overexpressing JDP2 for 5 weeks (n = 5) (Fig. 5). An increased susceptibility to apoptosis was reflected in the enhanced expression of bax mRNA (2-times higher in JDP2 mice vs. WT, n = 8, p ≤ 0.05) and a decrease in bcl2 mRNA (0.2-times lower in JDP2 mice vs. WT, n = 8, p ≤ 0.05). Also on the protein level an increase in Bax expression to 198 ± 38% (n = 8, p ≤ 0.05 vs. WT) and a decrease in Bcl2 expression to 13 ± 4% (n = 8, p ≤ 0.05 vs. WT) was observed.

An increase in heart weight was detected in JDP2-overexpressing mice with 146 ± 24 mg in WT mice vs. 165 ± 19 mg in JDP2 mice (n = 15–22, p ≤ 0.05). Body weights of WT mice (24.9 ± 5.4 g) was not different to JDP2 mice (24.4 ± 3.2 g, n = 15–22, n.s.). When determining heart to body weight ratio the enlargement of JDP2 hearts was also visible (6.0 ± 0.8 in WT mice vs. 6.8 ± 0.8 in JDP2 mice, n = 15–22, p ≤ 0.05) (Fig. 5). In addition, mRNA expression of ANP, as hypertrophic marker gene, was 26 times higher in JDP2 mice (n = 8, p ≤ 0.05 vs. WT mice).

**Figure 6:**
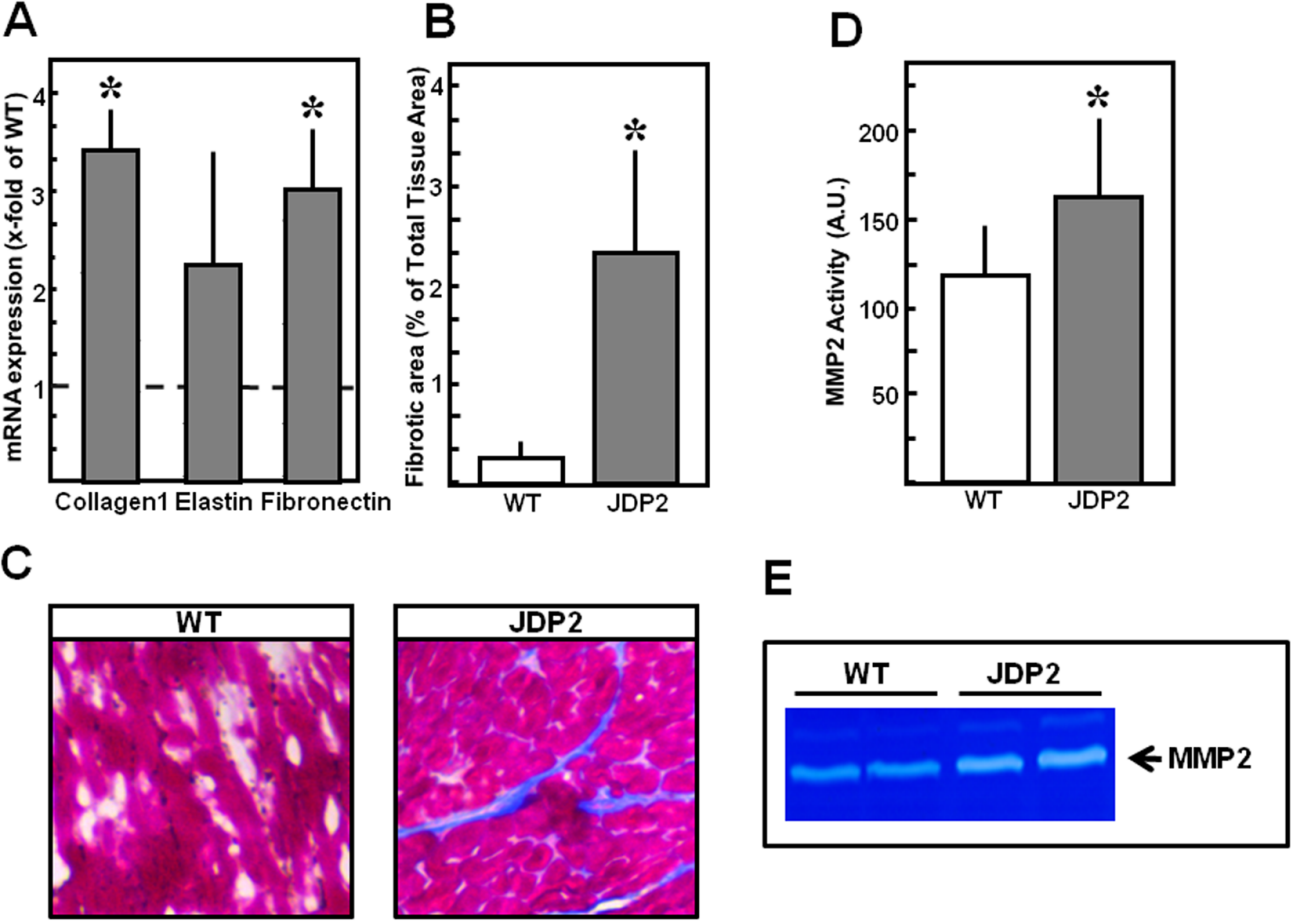
Fibrosis and MMP2 are enhanced in JDP2 mice. Hearts were extracted from mice five weeks after start of JDP2 overexpression and from age matched WT animals. **(A)** Fibrotic marker gene expression (n = 8). **(B)** Quantification of fibrotic area in percent to total tissue area (n = 6). **(C)** Tissue sections stained with azan dye reveal blue coloured fibrotic areas. **(D)** Densitometric analysis of MMP2 bands from zymograms (n = 7–12). **(E)** Representative zymogram. Data are means ± SD. *Differences from WT animals with p ≤ 0.05.

Furthermore, fibrosis was evaluated in mice overexpressing JDP2 for five weeks. Collagen1 and fibronectin mRNA were 3-times higher in JDP2 mice (n = 8, p ≤ 0.05 vs. WT, Fig. 6A). Increased expression of collagen 1 was confirmed on the protein level (224.8 ± 117.5% in JDP2 mice vs. WT, p ≤ 0.05, n = 12). Azan staining of ventricular tissues revealed enhancement of interstitial fibrotic areas (2.5 ± 1.1% vs. 0.2 ± 0.1 in WT animals, p ≤ 0.05, n = 6) (Fig. 6B,C). Going along with this, MMP2 activity is enhanced, thereby indicating occurrence of extracellular matrix remodeling under JDP2 overexpression (Fig. 6D,E).

In order to obtain indications of altered signaling pathways in JDP2 mice, phospho-kinase array was performed. Interestingly, only a small number of proteins showed altered phosphorylation patterns. Out of the 43 analysed proteins, PDGF-receptor β was the only protein with a significant increase in phosphorylation to 129.6 ± 16.2% in JDP2 mice (n = 6, p ≤ 0.05 vs. WT). This indicates activation of PDGF-signaling, which is a central mediator of tissue fibrosis^14^.

To determine if inflammatory processes occur in JDP2 mice, mRNA expression of inflammatory marker genes was evaluated by real time RT-PCR. In mice overexpressing JDP2 for five weeks TNFα mRNA increased 2.3-fold and interleukin1β mRNA (IL1β) 2.1-fold (p ≤ 0.05 vs. WT, n = 8).

## Discussion

The main finding of this study is that JDP2 overexpression in adult mice hearts is detrimental for heart function. Development of myocardial hypertrophy, fibrosis and cardiac dysfunction could be observed under JDP2 overexpression.

Previous descriptions of heart-specific overexpression of JDP2 in transgenic mice showed atrial hypertrophy and defects in the conduction system, but no overt ventricular phenotype was detected^12^. These studies were performed in 4 week old mice under continuous JDP2 overexpression. In our study, we now initiated JDP2 overexpression in the age of 4 weeks. Already one week after JDP2-overexpression impairment of ventricular function became evident, which was aggravated by extension of JDP2 overexpression to five weeks. Besides this impairment in ventricular function, atrial dilatation, ventricular hypertrophy, and fibrosis became evident in mice overexpressing JDP2 for 5 weeks. Main difference between our study and the one of Kehat *et al*.^12^, is the starting point of JDP2 overexpression, thereby indicating that JDP2 expression in juvenile mice is detrimental only in the atria, whereas in adult mice both atria and ventricles are affected. The occurrence of atrial dilatation in both studies (juevenile and adult mice), but induction of ventricular dysfunction only in adult mice, indicates that the atrial impairments are not the cause for development of ventricular dysfunction in adult animals. This is furthermore supported by our findings that ventricular dysfunction already develops in the first week of JDP2 overexpression. In addition, cardiomyocytes isolated after only one week of JDP2 overexpression, have a limited contraction capacity. This initial impairment of contractile function in ventricular cardiomyocytes may be the main reason for the reduced ventricular function of JDP2 mice *in vivo*. This conclusion is also supported by our own previous findings in isolated cardiomyocytes of JDP2 overexpressing mice, where we have shown that induction of JDP2 after birth impairs contractile function of cardiomyocytes within 7 weeks^10^. In the same study protective effects of JDP2 overexpression against induction of apoptosis or hypertrophy in isolated cardiomyocytes were found. However, no *in vivo* analysis on heart function of JDP2 overexpressing mice was performed at that time. This was made up for in the present study, revealing ventricular dysfunction in JDP2 mice already within one week of JDP2 overexpression. Combining the results of both studies, it can be assumed that the contractile dysfunction of cardiomyocytes plays a prevailing role in the phenotype in JDP2 mice and provokes ventricular dysfunction *in vivo*. The protective effects against hypertrophy and apoptosis which were found in isolated cardiomyocytes^10^ appear to play a subordinate role that does not work anymore under the long lasting ventricular dysfunction.

Moreover, mice overexpressing the JDP2 homolog ATF3 also develop cardiac hypertrophy and dysfunction in absence of atrial defects^15^. In ATF3 mice, cardiomyocyte-macrophage cross talk seems to play a major role in the adverse cardiac effects of ATF3^16^. In our study we found induction of inflammatory marker genes in JDP2-overexpressing mice. Therefore, the activated immune system may contribute to heart failure progression in JDP2 mice.

Factors that are increased during JDP2 overexpression are TNFα and IL1β. Both of these cytokines can upregulate and activate MMPs that are responsible for collagen degradation and subsequent matrix remodeling^17^. IL1β is also a predominant factor for collagen deposition in cardiac remodeling. It is not only released from cardiomyocytes but also from macrophages and endothelial cells resulting in a cytokine burst that initiates a vicious cycle of stress contributing to adverse cardiac remodeling. Also in our model, we identified activation of MMP2, enhanced collagen expression and ventricular fibrosis. MMP2 activation surely results in extensive extracellular matrix remodeling which is the primary determinant of passive left ventricular properties and plays a key role in force transmission and heart failure progression^18^. Another sign of ongoing fibrotic processes in JDP2 mice is the activation of PGFRbeta, since PDGF signaling is one of the central mediators in organ fibrosis^14^.

Interestingly, in our phospho-kinase array analysis PDGFRbeta was the only protein out of 43 that showed enhanced phosphorylation and thus activation in JDP2 mice. However, as we determined phospho-kinase levels at one time point only, we cannot exclude that other kinases were activated at earlier time points, and may also contribute to development of ventricular dysfunction.

Besides extracellular matrix remodeling and fibrosis induction, suppression of bcl2 expression was observed, and a trend to increased apoptotic levels. This potential loss of cardiomyocytes may additionally contribute to the reduced cardiac function. This result is consistent with the finding that the lack of JDP2 expression in neutrophils resulted in impaired apoptosis due to enhancement of bcl2 expression^19^.

Quite puzzling to our findings of impaired cardiac function under JDP2 overexpression is the fact, that knock out of JDP2 also provokes cardiac dysfunction in response to pressure overload^11^. However, these findings might mirror the divergent actions of JDP2: On the transcriptional control of gene expression JDP2 acts as specific AP-1 inhibitor but also as modulator of chromatin remodeling. And on the cellular level, JDP2 promotes contractile dysfunction but also impairs hypertrophy or apoptosis in cardiomyocytes^10^. In the study of Kalfon *et al*.^11^ JDP2 knock out mice basically presents normal cardiac function. Only after aortic constriction, stronger cardiac impairments became evident in JDP2 knock out mice compared to WT animals. Under aortic constriction, the loss of anti-hypertrophic and anti-apoptotic actions of JDP2 might be missing in order to prevent pressure overload induced damage. In contrast to JDP2 knock out, JDP2 overexpressing mice develop cardiac dysfunction in absence of any additional pathological stimulus. In this situation the leading role of JDP2 is the impairment of contractile function.

Interestingly, in humans enhanced expression of JDP2 after myocardial infarction correlated with HF progression^2^. This indicates that under this situation, the unfavorable function of JDP2 prevails and contributes to heart failure progression. However, as Maciejak *et al*.^2^ analysed JDP2 expression in PBMCs and we used cardiac-specific JDP2 overexpression, it remains to be determined, if JDP2 expression is increased also in cardiomyocytes after myocardial infarction to directly interfere with heart function in this setting.

In conclusion, the main finding of this study is that JDP2 overexpression in mice results in premature heart failure development. Thus, the promising protective effects against induction of hypertrophy and apoptosis, which were found in ventricular cardiomyocytes isolated from JDP2-overexpressing mice, could not be established *in vivo*. In contrast, JDP2 even has a detrimental character for heart function.

## Materials and Methods

The investigation conforms to the Directive 2010/63/EU of the European Parliament. Use of animals was registered at the Justus-Liebig-University (registration-no.: 419-M and 469_M). Animal studies were approved by the Regierungspräsidium Gießen (registration-no. V54–19c20–15(1) GI20/1 Nr. 54/2008 and V54 – 19 c 20 15 h 01 GI 20/1/ Nr. 57/2014).

### JDP2-overexpressing mice

JDP2 mice, crossbreeding of C57BL/6 J and FVB/N mice, had been generated in Haifa, Israel. They are double-transgenic, carrying a heterocygote JDP2 gene (tetO) with a minimal promoter and a heterozygote transactivator gene (tTA) under control of the cardiac-specific α-MHC promoter. The transactivator can be regulated by the antibiotic doxycycline (Dox) in a Tet-off system. Feeding of animals with Dox blocks the interaction between the transactivator and the promoter of the JDP2 gene, thereby preventing JDP2 overexpression. To suppress JDP2 overexpression during embryonic and juvenile development of mice, breeding pairs and newborn mice were fed with Dox until four weeks after birth. Then mice were fed with Altromin-standard diet for upto 5 weeks resulting in JDP2 overexpression (Fig. 1). Genotypes of JDP2 mice were tTA/tetO. As control, littermates of transgenic mice, which did not overexpress JDP2, (wild types, WT) were used. Thus, the WT group included the following genotypes 0/0, tTa/0 and tetO/0. WT mice received Dox-diet for the same times as the JDP2 mice.

### Blood pressure determination

Blood pressure was measured in conscious mice using a blood pressure monitor from TSE Systems GmbH (Bad Homburg, Germany). Mice were set in a restrainer (60 mm diameter) which was placed on a 35 °C warm heating plate in order to ensure sufficient blood flow in the tail. A tail-cuff was placed to occlude blood flow and a volume pressure sensor probe was placed distally to measure blood pressure and heart rate.

### Echocardiography

Cardiac function was assessed by transthoracic echocardiographic examination. Echocardiography was performed as described previously^20^. Mice were anesthetized with isoflurane (5% for induction and 2% for maintaining anesthesia), the chest was shaved, and the animal was placed in supine position onto a heating pad. Two-dimensional and M-mode echocardiographic examinations were performed in accordance with the criteria of the American Society of Echocardiography with a Vivid 7 Dimension ultrasound system (GE Healthcare, Global Operations Center, Budapest, Hungary) using a linear array 5.8–14 MHz transducer. Data of three consecutive heart cycles were analysed (EchoPac Dimension software; GE Healthcare, Global Operations Center, Budapest, Hungary) by an experienced investigator in a blinded manner. The mean values of three measurements were calculated and used for statistical evaluation. Systolic and diastolic anterior and inferior wall thickness as well as left ventricular end systolic and end diastolic diameters were obtained from parasternal short-axis view at the level of the papillary muscles. Systolic and diastolic septal and posterior wall thickness as well as left ventricular end systolic and end diastolic diameters were obtained from parasternal long-axis view at the level of the mitral valve. Left ventricular wall thicknesses were measured by means of M-mode echocardiography from short-axis and long-axis views between the epicardial and endocardial borders. Left ventricular diameters were measured by means of M-mode echocardiography from short-axis and long-axis views between the endocardial borders. Functional parameters including left ventricular end-diastolic volume, left ventricular end-systolic volume, and ejection fraction were calculated on short-axis view images according to the formula: EF (%) = (LVEDD^3^-LVESD^3^)/LVEDD^3^ × 100^21^. For analysis of ventricular functions in the 1 week group and of atrial dimensions echocardiography was performed with Vevo 2100 imaging systems (Visualsonics, Toronto, Canada) equipped with 18 to 38-MHz (MS400, mouse cardiovascular) transducer. Atrial area was measured at the apical four-chamber view. Clear visualization of both mitral and tricuspid valves movement was accounted as a correctly acquired position for measurement of the end-diastolic LA area.

### Cell isolation and culture

Mice were anaesthetized by isofluran inhalation. After cervical dislocation hearts were extracted, and retrograde-perfused in a Langendorff apparatus with a collagenase-containing calcium-free buffer equilibrated at 37 °C, pH 7.4. After separation of cardiomyocytes from other cardiac cells by centrifugation, medium was readjusted to a physiological calcium concentration and suspended in basal culture medium. Cardiomyocytes were then plated on laminin coated culture dishes. After 2 hours medium was changed and cells could be stimulated. The basal culture medium (CTT) was modified medium 199 including Earl’s salts, 2 mM L-carnitine, 5 mol/L taurine, 100000 IU/L penicillin, 100 mg streptomycin and 10 μmol/L cytosine-beta-D-arabinofuranoside.

### Cell contraction

Cell shortening was analysed as described previously^13^. Briefly, cardiomyocytes were stimulated at 2 Hz for 1 min at room temperature for analysis of basal contraction or under incubation with 10 nM isoprenaline. Analysis of cell contraction was performed using cell-edge detection system. Cells were stimulated via two AgCl electrodes with biphasic electrical stimuli composed of two equal but opposite rectangular 50 V stimuli of 5 ms duration. Only rod-shaped cells that contracted regularly during the whole time of measurement were used. Every 15 s cell shortening, contraction and relaxation velocities were measured using a line camera. The mean of four measurements per cell was used as average value of each individual cardiomyocyte. Cell shortening data were normalized to the individual diastolic cell length (dL/L %).

### Isolation of Hearts

Mice were committed to euthanasia by isoflurane inhalation (5% isoflurane) and cervical dislocation. Hearts were directly excised and blood was washed out with ice-cold 0.9% NaCl. Afterwards tissue was either shock frozen in liquid nitrogen and stored at −80 °C or frozen tissue sections were made.

### Real time RT-PCR

Total RNA from left ventricles was extracted with Trizol (Invitrogen) as described by the manufacturer. This was followed by DNAse treatment and reverse transcription with QuantiTect Reverse Transcription Kit from Qiagen. For each assayed gene, annealing temperature and the number of cycles resulting in a linear amplification range were tested. Real time RT-PCR was performed in an automated thermal cycler and was detected with the Biorad detection system (Biorad) using SYBR Green fluorescence for quantification. The calculations of the results were carried out according to the 2^−ΔΔCt^ methods as described^22^. Gene expression was related to hypoxanthine phosphoribosyltransferase (HPRT) as housekeeping gene. Primer sequences are listed in Table 2.

**Table 2:** Primer sequences for real time RT-PCR.

ANP	5′-CTGCTAATCAGCCATGCAAA-3′ 5′-GATGGAGACCATCCTGGCTA-3
Bax	5′-ACTAAAGTGCCCGAGCTGATC-3′ 5′-CACTGTCTGCCATGTGGGG-3′
Bcl2	5′-GGGAGAACAGGGTATGATAAC-3′ 5′-AGGCTGGAAGGAGAAGATG-3′
Collagen1	5′-TTCTCCTGGRAAAGATGGTGC-3′ 5′-GGACCAGCATCACCTTTAACA
Elastin	5′-CTGCTGCTAAGGCTGCTAAG-3′ 5′-CCACCAACACCAGGAATGC-3′
Fibronectin	5′- ACAGAGCTCAACCTCCCTGA-3′ 5′-TGTGCTCTCCTGGTT CTCCT-3′
HPRT	5′-CCAGCGTCGTGATTAGCGAT-3′ 5′-CAAGTCTTTCAGTCCTGTCC−3′;
Interleukin1β	5′-GAAATGCCACCTTTTGACAGTG -3′ 5′-GTGCTGCTGCGAGATTTGAAG-3′
JDP2	5′-ATGATGCCTGGGCAGATCCCA-3′ 5′-TCACTTCTTGTCCAGCTGCTCC-3′
SERCA	5′-TGACTGGTGATGGTGTGAATG-3′ 5′-GATGAGGTAGCGGATGAACTG-3′
TNFα	5′-CCGATGGGTTGTACCTTGTC-3′ 5′-GGGCTGGGTAGAGAATGGAT-3′

### Immunoblot analysis

Proteins were extracted from frozen hearts. Hearts were homogenized in RIPA buffer (50 mmol/L Tris/HCl, pH 7.5, 150 mmol/L NaCl, 1% Nonidet P-40, 0.5% deoxycholat, 0.1% SDS, 1 mM PMSF, 1 mM EDTA, 1 mg/l pepstatin). Nucleic acids were digested with benzonase. Samples were denatured in Laemmli buffer at 90 °C for 5 min, loaded on 12.5% SDS-gels, and blotted on PVDF membranes. For detection of JDP2 expression, HA-antibodies (Bethyl) were used, and for loading controls, vinculin antibodies (Sigma). Protein bands were detected by horseradish peroxidase-labeled secondary antibodies using ECL as detection system (Pierce).

### Phospho-kinase array

For detemination of kinase phosphorylation patterns in cardiac protein extracts we used Human phospho-kinase array kit form R&D systems. This kit enables detection of relative levels of phosphorylation of 43 kinase phosphorylation sites and 2 related total proteins. The array was used as described by the company.

### Frozen Tissue sections

Whole ventricles are embedded in “Tissue Freezing” (R. Langenbrinck) on Cryomold bowls (Weckert Labortechnik). Then sample are snap-frozen in 2-Methylbutane-isopentan (Fluka). Frozen tissues are cut into slices and used for determination of fibrosis or apoptosis (see below).

### TUNEL Assay

After digestion of frozen tissue sections with proteinase K, samples were labeled with biotin-labeled dUTP using terminal transferase. Apoptotic nuclei became visible by streptavidin texas red staining.

### Fibrosis Assay

Frozen tissue sections were stained with hematoxylin-eosin to detect muscle tissue in red and azan dye to detect collagen in blue. Micrographs were taken, and fibrotic areas were determined using Image J software.

### Zymography Assay

Gelatinolytic activities of MMP-2 were examined as previously described in detail^23^. Briefly, 8% polyacrylamide gels, co-polymerized with gelatin (2 mg/ml) were loaded with 50 µg of protein. After electrophoresis, gels were washed with renaturation buffer (containing 2.5% Triton X-100), and then incubated in development buffer to eliminate Triton-X-100. Gels were stained with 0.05% Coomassie brilliant blue, and gelatinolytic activities were detected as transparent bands against the dark-blue background. Band intensities were quantified (Quantity One software, Bio-Rad,Hercules, CA) and expressed in arbitrary units.

### Statistics

Data are given as mean ± standard deviation from n different animals. Statistical comparisons were performed by ANOVA (One-Way Analysis of Variance) and Student-Newman-Keuls test or student-T-test. A p-value of less than 0.05 was considered statistically significant.

### Data availability

The datasets generated during and/or analysed during the current study are available from the corresponding author on reasonable request.
